# Speaker and Accent Variation Are Handled Differently: Evidence in Native and Non-Native Listeners

**DOI:** 10.1371/journal.pone.0156870

**Published:** 2016-06-16

**Authors:** Buddhamas Kriengwatana, Josephine Terry, Kateřina Chládková, Paola Escudero

**Affiliations:** 1 Institute for Biology Leiden, Leiden University, Leiden, the Netherlands; 2 Department of Psychology, University of Amsterdam, Amsterdam, the Netherlands; 3 The MARCS Institute for Brain, Behaviour, and Development, Western Sydney University, Sydney, Australia; 4 ARC Centre of Excellence for the Dynamics of Language, Australian National University, Canberra, Australia; 5 Amsterdam Center for Language and Communication, Phonetic Sciences, University of Amsterdam, Amsterdam, the Netherlands; 6 Institute of Psychology, University of Leipzig, Leipzig, Germany; Leiden University, NETHERLANDS

## Abstract

Listeners are able to cope with between-speaker variability in speech that stems from anatomical sources (i.e. individual and sex differences in vocal tract size) and sociolinguistic sources (i.e. accents). We hypothesized that listeners adapt to these two types of variation differently because prior work indicates that adapting to speaker/sex variability may occur pre-lexically while adapting to accent variability may require learning from attention to explicit cues (i.e. feedback). In Experiment 1, we tested our hypothesis by training native Dutch listeners and Australian-English (AusE) listeners without any experience with Dutch or Flemish to discriminate between the Dutch vowels /I/ and /ε/ from a single speaker. We then tested their ability to classify /I/ and /ε/ vowels of a novel Dutch speaker (i.e. speaker or sex change only), or vowels of a novel Flemish speaker (i.e. speaker or sex change plus accent change). We found that both Dutch and AusE listeners could successfully categorize vowels if the change involved a speaker/sex change, but not if the change involved an accent change. When AusE listeners were given feedback on their categorization responses to the novel speaker in Experiment 2, they were able to successfully categorize vowels involving an accent change. These results suggest that adapting to accents may be a two-step process, whereby the first step involves adapting to speaker differences at a pre-lexical level, and the second step involves adapting to accent differences at a contextual level, where listeners have access to word meaning or are given feedback that allows them to appropriately adjust their perceptual category boundaries.

## Introduction

**Fig 1:**
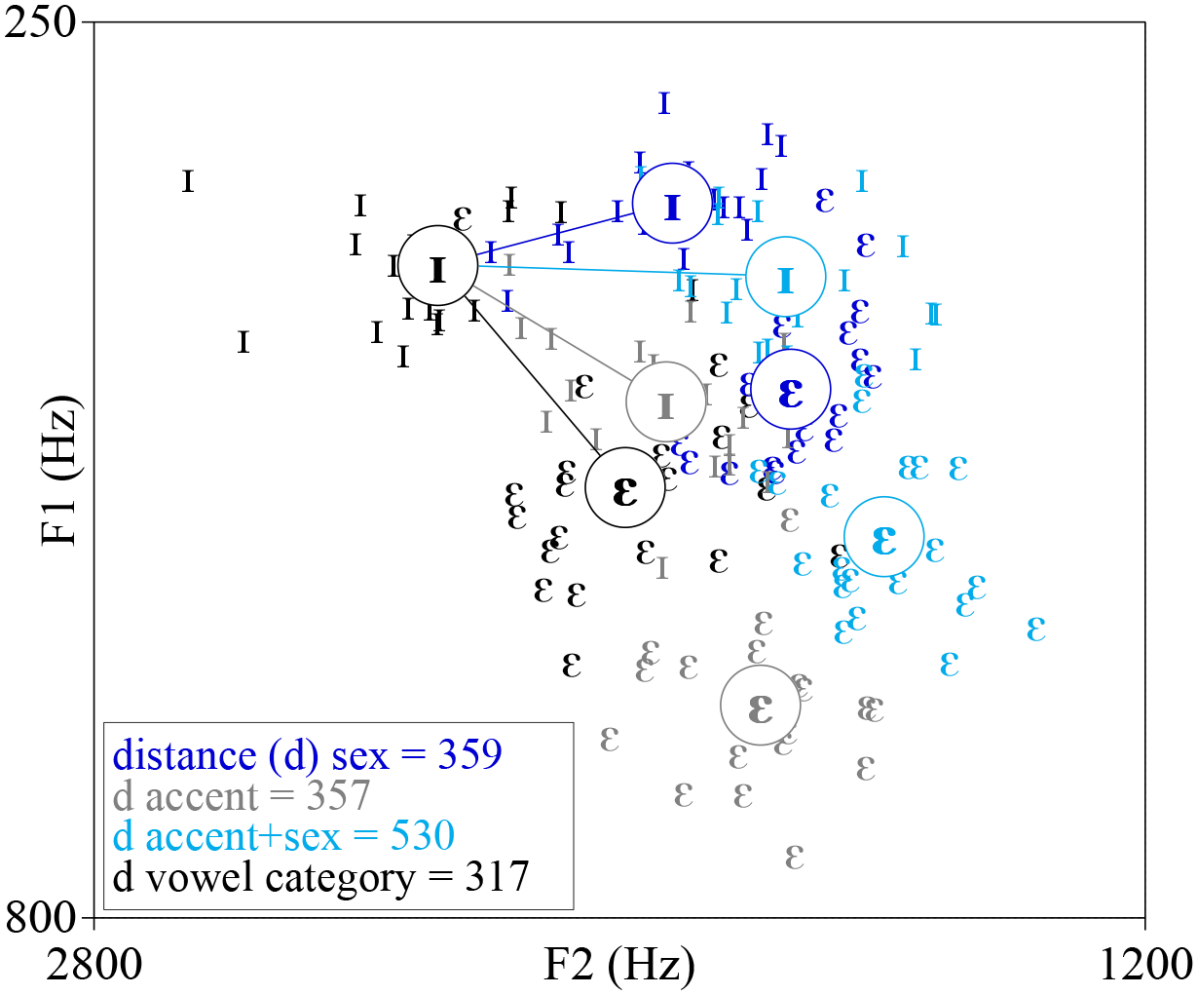
F1-F2 plot of /I/ and /E/ tokens produced by 40 speakers of North-Holland Dutch and West Flemish accents (two tokens per speaker) from Adank et al. (2004). These tokens are the stimuli used in our experiments. Black = Female Dutch, grey = Female Flemish, Dark blue = Male Dutch, Light blue = Male Flemish. The average values for each group are circled. Note that there is variation between speakers of the same sex, speakers of the opposite sex, and speakers of the same accent (as well as between accents). The average acoustic distance between vowel category, sex, accent, and Accent+Sex is shown by lines connecting the respective symbols and listed in the left bottom corner. This shows that acoustic distance in F1-F2 space (computed as the Euclidean distance in Hz) does not reliably distinguish between different types of variability, especially sex and accent, as their distance is almost identical.

It is remarkable that even though different speakers produce the same speech sound differently, listeners are still able to reliably identify that sound. How this is achieved has long been a topic of great scientific interest, especially because listeners have to cope with different types of speaker-related variation. The acoustic properties of vowels provide compelling evidence of this problem: the same vowel produced by different speakers varies tremendously, especially if speakers are of different ages, sexes, and sociolinguistic backgrounds (e.g. [1,2]). We illustrate both types of variation (i.e. speaker/sex variability and accent variability) with Dutch and Flemish vowels (Fig 1), where F1 and F2 correspond to the first and second formant frequencies (i.e. resonant frequencies of vocal tract that are modulated by the position of the tongue and by the shape and degree of opening of the lips) that are commonly used for vowel identification.

Variation between speakers in the production of vowels is generated primarily from two sources: morphological sources and sociolinguistics sources (e.g. [1,3,4–6]). Sex differences in the length of the vocal cords in the larynx (source) and supralaryngeal vocal tract length (filter; e.g. [7]) between men and women contribute to differences in acoustic signal that signify speaker sex (a highly salient indexical feature in speech) and can cause the same vowel produced by different speakers to differ in its resonant frequencies, most notably the first and the second formant frequency (F1 and F2). For instance, the vowel in the English word *seat* has an F1 of about 342 Hz when it is produced by an average male speaker and 437 Hz when it is produced by an average female speaker; or, the vowel in *sit* has an F1 of about 427 Hz when it is produced by an average male speaker and 483 Hz when it is produced by an average female speaker [2]. This example illustrates that the absolute formant differences between (some) male and female vowels are comparable to a categorical/phoneme difference produced by speakers of the same sex. The larger variation that is typically observed in F1 and F2 values between speakers of different sexes (compared to speakers of the same sex) can be largely attributed to sex differences in vocal tract morphology. Sociolinguistic factors include the speech characteristics of the listener’s linguistic community, cultural stereotypes of gender-typical speech, and cultural expectations of politeness. These factors are an important source of speaker variation because they can influence speakers’ phonetic realizations (e.g. [4,8,9]), and play an important role in influencing vowel realizations, especially in terms of influencing accent differences in vowel production [2]. Accent differences are likely due to source and filter properties related to speaker differences (which have been found to be a function of individual differences in vocal tract transfer function [10]), but may additionally differ in the mapping of formant frequencies to phonetic categories. Note that between-speaker variation within an accent also exists: speakers of the same accent also show differences in vowel production, however, variation within accents are smaller than variation between accents (see Fig 1 for a sample distribution of speaker, sex, and accent differences in vowel production). Thus, morphological factors related to the vocal apparatus systematically contribute to speaker and sex differences while sociolinguistic factors contribute greatly to accent differences.

The F1 and F2 of vowels are acoustic properties important for vowel identification [1–3, 5,11]. Recall that the F1 of the vowel in *seat* differs between male and female speakers; crucially the *seat*-vowel produced by women is more similar to the male-produced *sit*-vowel than to the male-produced *seat*-vowel. Nonetheless, the variability in vowels’ acoustic properties caused by individual differences between speakers does not seem to hinder correct vowel identification by listeners. Different explanations of how listeners adapt to acoustic variability between speaker fall into two broad classes: exemplar-based theories and perceptual normalization theories. Exemplar-based theories suggest that listeners adapt to speaker differences by comparing each speech sample to a set of exemplars that they have previously encountered, and classify samples based on their resemblance with these stored exemplars. Exemplars retain all acoustic information (linguistic and non-linguistic) available at the time they were encountered and connect a set of auditory properties to a set of category labels (e.g. vowel category, speaker identity, speaker sex and speaker accent). On the other hand, perceptual normalization theories assume that listeners deal with speaker variability by transforming and reducing the acoustic speech signal into particular components that map onto invariant phonological representations [12]. This normalization process that transforms the acoustic signal serves to minimize acoustic variability of members within a phonetic category while maximizing differences between phonetic categories (reviewed in [11,13–15]). Different vowel normalization procedures have been put forth and tested, with varying degrees of effectiveness at normalizing speaker and sex differences in vowel production (e.g. [14,16]).

**Fig 2:**
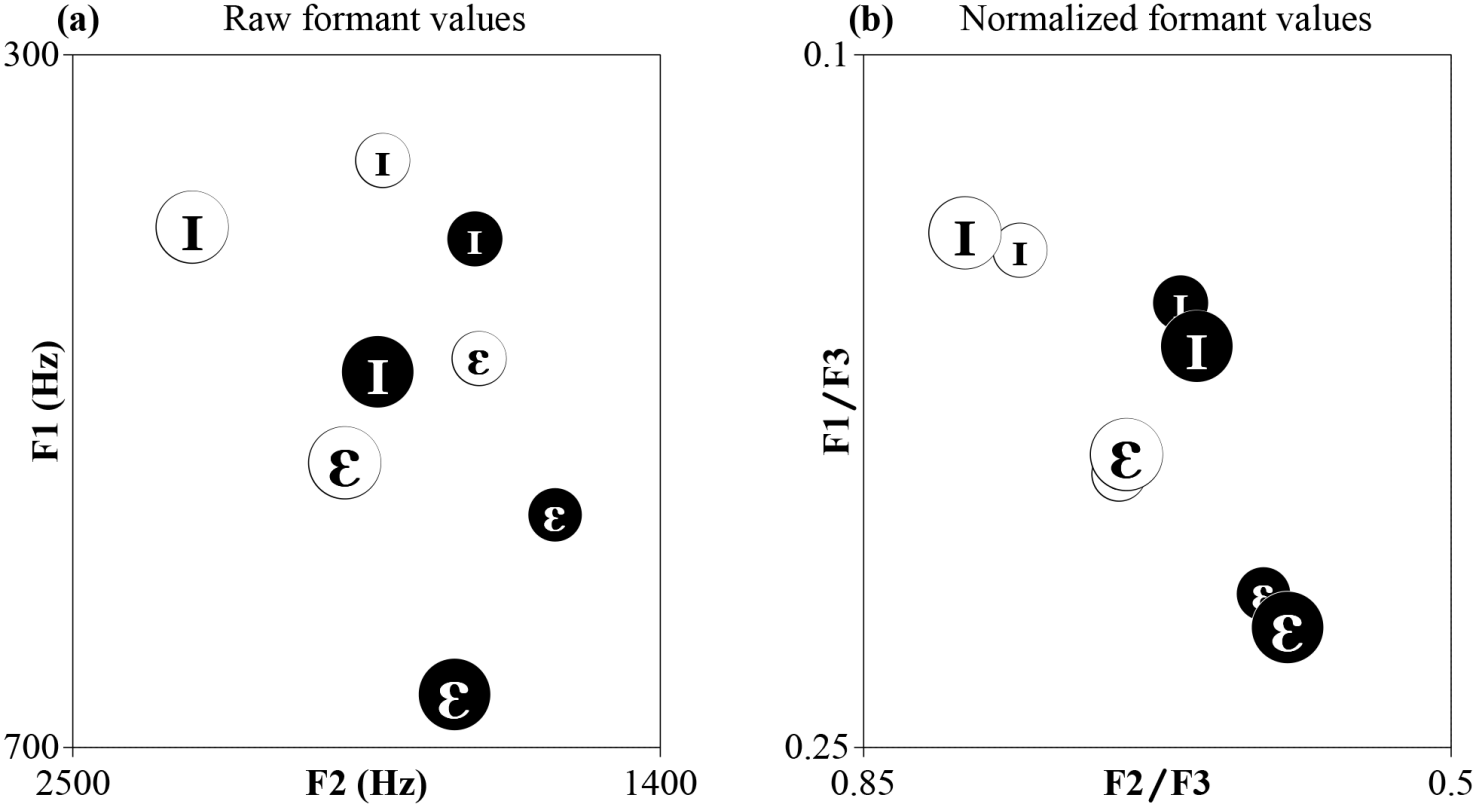
(a) F1 and F2 values of the average Dutch (black symbols in white circles) and average Flemish (white symbols in black circles) vowel tokens used in the present study; (b) formant ratios of the stimuli in (a). Female tokens are plotted in larger font and larger circles. The figure shows average formant values across several tokens that were used in the experiments (namely, across 8 tokens for Speaker change, and 12 tokens for each Sex, Accent, and Accent+Sex change).

Adult speakers are also remarkably skilled at adapting to accent differences (see [17, 18]). However, there is currently no consensus about whether accent variation is a type or speaker variation, or a different type of variation entirely, which means there is also no agreement on whether listeners handle speaker and accent variation in the same way. Exemplar-based theories seem to view accent variation as similar to speaker variation because detailed representations of linguistic and non-linguistic information contained in speech samples are stored. Both speaker and/or accent adaptation occurs when incoming speech samples are matched with stored exemplars of similar-sounding speakers that the listener has previously encountered. Proposals for speech normalization procedures have not made explicit predictions about whether listeners adapt to speaker and accent variability similarly or differently. But if speaker and accents are different types of variation that are handled by different processes, we might expect that normalization procedures would not be effective at reducing variation in accents because they were developed with the purpose of reducing variation stemming from morphological characteristics of the speaker, such as vocal tract length (e.g. [11,19–21]). An example of the effectiveness of a vowel normalization procedure that transforms formant frequencies into formant ratios F1/F3 and F2/F3 [22] is given (Fig 2). This transformation procedure dramatically reduced speaker/sex differences in a corpus of American English vowels [22], and also minimized variation caused by speaker/sex differences in the Dutch and Flemish vowels used in our study. However, as we predicted, this formant ratio transformation is noticeably unsuccessful at reducing variation between Dutch and Flemish accents (Fig 2). Consequently, if listeners normalize vowels by automatically transforming formant frequencies into formant ratios [22], then it seems plausible that distinct normalization mechanisms handle speaker compared to accent variation.

We hypothesize that listeners adapt to speaker/sex and accent variation differently, as has been alluded to previously (e.g. [17,23]). The main reason for our hypothesis is that speaker/sex variation and accent variation stem from different sources. Both anatomical and sociolinguistics factors can contribute to speaker/sex variation, whereas anatomical factors are unlikely to significantly contribute to meaningful variation in accented speech. Adapting to speaker/sex differences is required for all speakers while adapting to accent differences is required only for specific groups of speakers. Therefore, we reason that listeners can adapt to speaker/sex variability pre-lexically, but that they require additional contextual cues (possibly via the form of listener expectations, lexical knowledge, or perceptual learning) to initiate distinct mechanisms to adapt to accent variability [12,13,18]. Consequently, we postulate that adapting to accents may be a two-step process, whereby the first step involves normalization of speaker differences at a pre-lexical level, and the second step involves normalization of accent differences at a contextual level (i.e. using feedback from lexical, semantic, or other cues to adjust perceptual categories if needed). This idea of multiple stages in phonemic perception is not unprecedented: for instance, Escudero & Bion [16] presented a computational model where vowel classification is a two-step process involving pre-lexical normalization of vowels and subsequent linguistic classification that acts on normalized values.

Several studies with children, adults, and non-human animals provide support for the claim that adapting to speaker/sex differences are guided by pre-lexical mechanisms. First, pre-lexical infants can adapt to speaker/sex differences but not accent differences when discriminating vowels [24,25]. Second, studies in adults suggest that processing of vowel acoustics is pre-attentive and occurs at low levels of processing [26,27], and that detection of a different accent recruits additional unique brain regions involved in semantic processing compared to detection of a different speaker [28]. Third, various non-human animals can classify words and isolated vowels despite speaker and sex differences [29–34] (for a review see [35]), and can do so even after experience with only a single speaker [35]. This suggests that dealing with speaker/sex differences in speech segments may to a certain degree rely on innate processes shared with other animals.

Evolutionary processes or extensive experience with constantly adapting to different speakers may explain why the automaticity of speaker adaptation is automatic in adults (i.e. we will always need to adapt to different speakers no matter what language we speak). On the other hand, it seems intuitive that processing of accented speech is not pre-lexical or automatic but instead requires learning of the idiosyncrasies in speech patterns associated with speakers with a particular accent [6,36]. This line of reasoning argues in favor of separate mechanisms for handling speaker differences and accent differences. However, current evidence cannot be used to falsify this hypothesized distinction because studies in accented speech have always used words or sentences, where information for adaptation is available for both speaker and accent changes (e.g. [36–42]). Words and sentences give participants access to linguistically relevant semantic and lexical cues, which may be crucial for accent adaptation because they provide listeners with feedback about the accuracy of their adaptation to the accented speech. If access to feedback regarding the accuracy of their adaptation (through lexical, semantic, or other contextual cues) is indeed necessary in order to adapt to accents, then removing feedback should significantly hinder accent adaptation. Conversely, removal of feedback regarding accuracy of adaptation should not affect speaker adaptation because cues for speaker identity and sex are present in very short speech segments [10], and listeners readily show speaker adaptation to isolated vowels without needing additional lexical or semantic information (e.g. [43–46]). In short, a different accent may be difficult to deduce based on acoustic-phonetic information alone whereas a different speaker or sex may not, which means that adaptation to accents and speakers may require different information and involve different processes.

The present study attempts to dissociate potential differences in processing of speaker and accent variation by testing listeners’ ability to adapt to isolated vowels of different speakers and accents when feedback on the accuracy of their adaptation is unavailable. We employed isolated vowels instead of words or sentences in order to control the presentation of contextual cues that trigger the activation of additional (linguistic) processes that we believe may be involved in compensation for accent differences. To test our hypothesis that speaker/sex adaptation occurs by default whereas accent adaptation is triggered only in the presence of additional cues that inform listeners about the accuracy of their adaptation in adult listeners, we trained adult Australian English (AusE) listeners to categorize /I/ and /ε/ vowels from one Dutch speaker (*FAM*), and then tested their ability to categorize the vowels of another speaker (*NEW*) with the same or different accent (Dutch or Flemish). Importantly, we either withheld (Experiment 1) or gave participants feedback (Experiment 2) about whether their categorizations of the *NEW* speaker’s vowels were correct or incorrect. Native Dutch-speaking participants were also tested to address whether the results obtained from AusE listeners were simply due to inexperience with Dutch and Flemish vowels. If both speaker and accent adaptation occur by default, then listeners will correctly categorize all *NEW* vowels when no feedback is given about the accuracy of their adaptation. However, if accent adaptation is triggered only in the presence of additional cues that inform listeners of the accuracy of their adaptation, then listeners will incorrectly categorize only accented *NEW* vowels when no feedback is given. In Experiment 2, we provided feedback on accuracy of adaptation by informing listeners whether categorization of *NEW* vowel tokens was correct or incorrect. Functionally, this kind of feedback informs listeners to adjust perceptual categories if necessary, and is constantly available in real life situations in the form of contextual, lexical, and/or semantic information. If accent adaptation is triggered by additional contextual cues, then feedback in Experiment 2 may improve listeners’ categorization performance.

To test our hypothesis, we used a Go/No-go classification paradigm. The Go/No-go task is an implicit measure of perceptual categorization that is widely used in studies of categorization and has been used previously to successfully test auditory and vowel categorization in humans (e.g. [47–49]). In the Go/No-go task, there are two types of trials: Go trials and No-go trials (e.g. /I/ and /ε/). On each trial, listeners hear a vowel and can decide to make a response by pressing a button (Go), or to not make a response by not pressing any buttons (No-go). There are two main reasons why the Go/No-go task was more suitable for our study compared to the other perceptual categorization tasks commonly used in psycholinguistic studies. First, the Go/No-go task ensures that listeners’ categorizations are not influenced by orthography, which has been demonstrated to play a role in non-native speech perception [50]. Second, vowel categories are not explicitly provided at the beginning of the experiment, so listeners must perceptually learn and distinguish the categories themselves during training trials. Therefore, we can be relatively certain that listeners’ responses to test trials provides a strong test of auditory category learning rather than auditory discrimination (which could be possible in other tasks, such as the XAB task, where listeners compare whether stimulus X sounds more like stimulus A or B). These two aspects of the Go/No-go task make it ideal for testing our hypothesis that speaker and accent adaptation are handled differently because we can confidently assume that listeners must adapt to speaker and accent differences in order to be able to correctly categorize the tokens from the second, new speaker in the Go/No-go task. Specifically, correct categorization of *NEW* vowels should demonstrate their ability to adapt to a different speaker or accent. We assume that when listeners make a Go response to a *NEW* token, it means that they think that the *NEW* token belongs to the same category as the *FAM* Go stimuli as opposed to the *FAM* No-go stimuli. Similarly, if listeners do not make a response to a *NEW* token (i.e. No-go), it suggests that they judge the *NEW* token as belonging to the same category as the *FAM* No-go stimuli. To determine whether listeners correctly adapted to speakers and/or accents, we measured: (1) whether they assigned *NEW* tokens to the correct categories (i.e. did they treat *NEW* Go similar to *FAM* Go, and *NEW* No-go similar to *FAM* No-go?); and (2) whether they could discriminate between the NEW tokens (i.e. *NEW* Go compared to *NEW* No-go).

## Experiment 1

### Materials and Methods

#### Participants

All participants were adults and provided written informed consent before participating in the experiments. The ethics protocols and written consent procedure for these experiments were approved by the Faculty of Humanities Ethics Committee (University of Amsterdam) and Human Research Ethics Committee (University of Western Sydney).

Eighty (63 females, 17 males) undergraduate students from the University of Western Sydney participated in the study in exchange for course credit (Mean age = 22.51 years, *SD* = 6.79, Range = 18–47 years). Twenty-eight participants were monolingual Australian English speakers. Forty-three participants spoke one language other than English (i.e. bilingual), and 9 spoke multiple languages other than English (i.e. multilingual). The additional languages spoken were Arabic (10), Assyrian (1), Bengali (2), Cantonese (1), Chaldean Neo-Aramaic (1), Dari (1), Egyptian (1), French (2), German (1), Greek (1), Hakka (1), Hindi (5), Indonesian (1), Iranian (1), Italian (2), Japanese (1), Khmer (2), Korean (1), Macedonian (1), Malayalam (1), Mandarin Chinese (5), Persian (2), Polish (1), Punjabi (1), Serbian (3), Somali (1), Spanish (1), Tagalog (1), Thai (1), Turkish (1), Urdu (3), and Vietnamese (6). The numbers of participants speaking each language are indicated in parentheses. Apart from three participants whose native language was Mandarin; all participants had English as their first language. None of the listeners had prior exposure to Dutch or to any Dutch accent.

Approximately half the participants (29 females, 11 males, Mean age = 21.90 years, *SD* = 6.41, Range = 18–47 years) completed the Speaker and Sex change conditions, while the remaining subjects (34 females, 6 males, Mean age = 23.13 years, *SD* = 7.17, Range = 18–46 years) completed the Accent and Accent+Sex conditions (as described in the Procedure section). The number of bilingual/multilingual listeners was roughly balanced across conditions: 29 in the Speaker/Sex conditions (11 monolinguals) and 23 in the Accent/Accent+Sex conditions (17 monolinguals).

Twenty-four native speakers of Dutch took part in the experiment (15 females, 9 males; mean age = 23.83, SD = 3.86, range = 19–35 years). They were students at the University of Amsterdam (North Holland, the Netherlands) and were paid for participation. They were all native speakers of Dutch as it is spoken in the Netherlands: 13 were raised in the Randstad area, 11 in other parts of the Netherlands; 19 indicated they had no accent or the ‘standard’ accent in Dutch, 5 noted they sometimes also used other Dutch accents (mostly when speaking with their parents). North Holland Dutch listeners often encounter Flemish Dutch via frequent exposure through media (radio and television) and in many cases via interactions with Flemish speakers. Because Dutch listeners were proficient in Dutch and English, we classified all Dutch listeners as bilinguals (although we acknowledge that they are not bilingual in the same sense as our AusE listeners because Dutch listeners used English only at University while our bilingual AusE listeners used English in other settings as well). This is in contrast with our bilingual AusE listeners, who used their native language at home but also had to use English on a daily basis to interact with others at the university and in all other settings outside of their homes. Analogously to the AusE participants, half of the Dutch participants completed the Speaker and Sex conditions, and the other half completed the Accent and Accent+Sex conditions. Please refer to S1 Data for the number of Dutch and AusE participants that were assigned to each stimulus set.

#### Stimuli

Stimuli were natural isolated Dutch and Flemish vowels /I/ and /ε/ produced by native Dutch and Flemish female and male speakers. The vowels were extracted from a syllable s-Vowel-s spoken in a carrier sentence /In sVs ən In sVsə zIt də V/ [51], which translates to “In X and in Y there is a Z”, where X, Y and Z are nonce words for both English and Dutch. Two tokens of each vowel from each speaker were extracted. We included tokens from several different speakers to ensure that the results we obtained were generalizable (i.e. not limited to idiosyncrasies related to a particular token). The naturally spoken extracted vowels were edited in the program Praat [52]. The vowels were scaled to have equal intensity and were ramped at their edges with 5-ms on- and off-glides, and their duration was manipulated to be approximately 60 ms by either copy-pasting or deleting periods from the central portion of the vowel.). The acoustic values of the vowel-tokens are presented in S1 Table and the mean values are plotted in Fig 1a. The auditory stimuli were presented at a comfortable listening level over Sennheiser HD280 circumaural headphones via an Edirol UA-25 USB audio interface.

**Fig 3:**
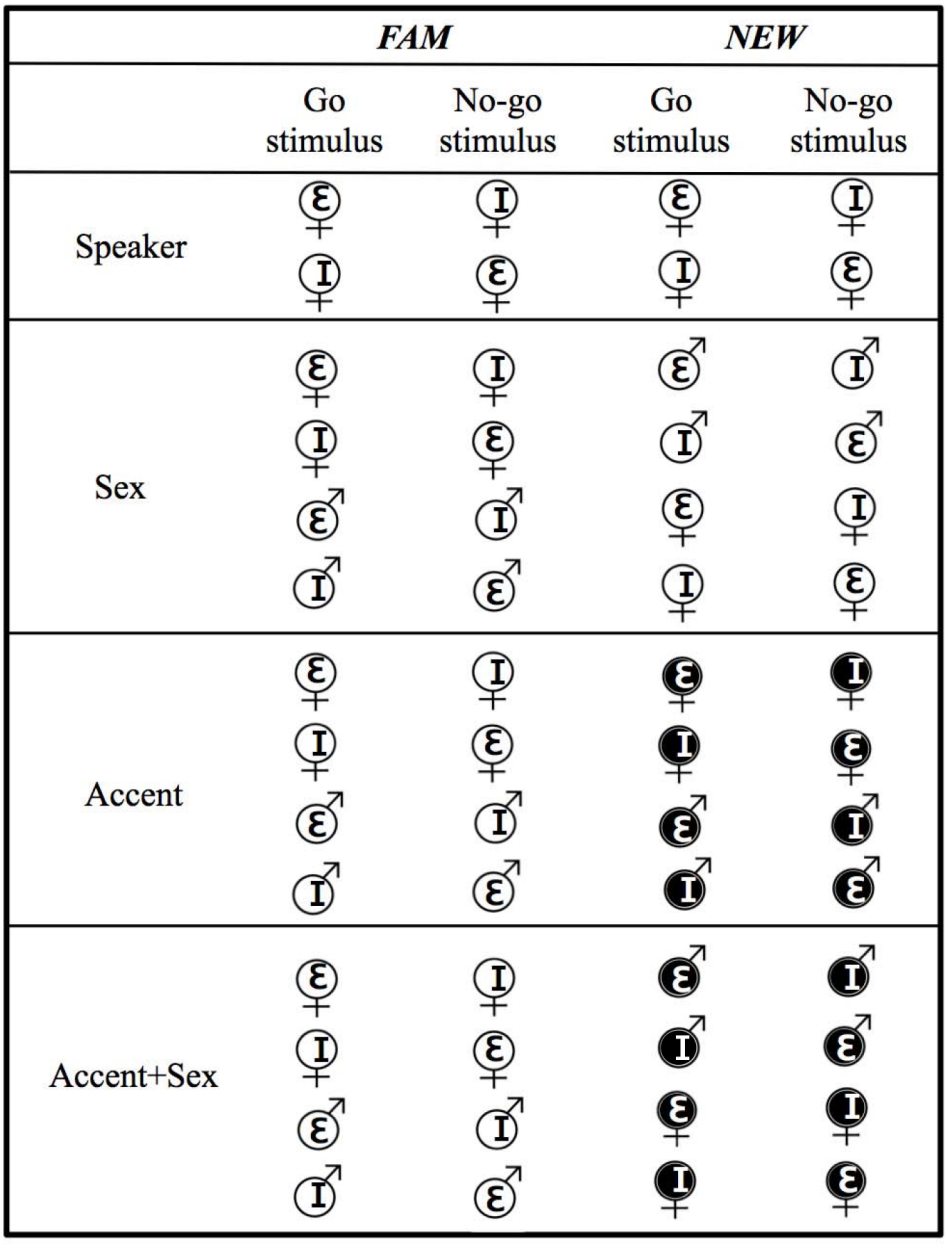
Stimulus sets used in Speaker, Sex, Accent and Accent+Sex conditions used in Experiment 1 and 2 (Dutch = black symbols in white circles, Flemish = white symbols in black circles). Each participant was given one set per condition, and tested in two conditions (either Speaker and Sex, or Accent and Accent+Sex).

Fig 3 illustrates the stimuli sets used in the Speaker, Sex, Accent, and Accent+Sex conditions. In total there were 4 stimuli sets in the Speaker condition containing four different speakers and 8 stimuli sets in the Sex, Accent, and Accent+Sex conditions each containing eight different speakers. Sets were counterbalanced for whether the /I/ or /ε/ vowel was the Go or No-go stimulus across participants. Many sets of stimuli were made to ensure that results we obtained were not due to idiosyncratic characteristics in our natural vowel stimuli. However, each listener heard only one set of stimuli per condition. Each stimulus set consisted of vowels from two different speakers. One speaker was presented during training, and the other was presented at test. Thus, each listener heard only two speakers (one *FAM* and one *NEW*) in each condition, and participated in two conditions (Speaker and Sex, or Accent and Accent+Sex).

#### Procedure

Participants completed two Go/No-go tasks within a single experimental session: either Speaker and Sex conditions, or Accent and Accent+Sex conditions. Assignment to these conditions was random, and the order to the two conditions was counterbalanced across participants. Each experimental session lasted one hour, with each condition taking 25 minutes to complete.

The Go/No-go tasks were conducted on a computer running E-prime version 2. Minimal instructions about the aims of the Go/No-go task were given to participants because we did not want to bias or influence listeners’ perceptual learning of vowel categories. Participants were informed that they would hear a sound and that they were to determine what was required to elicit a correct response. They were instructed to earn as many points as possible by making a correct response, and by avoiding an incorrect response. Points were awarded to motivate participants to try to make correct responses. In the Go/No-go task listeners can do two things after hearing the sound: they can choose to press a button (i.e. respond/Go) or to do nothing for 2 seconds (i.e. not respond/No-go). To make a correct response, participants had to press “spacebar” when presented with a stimulus from one of the two vowel-token sets (Go tokens). They had to withhold a response and not press any key when presented with a stimulus from the other vowel-token set (No-go tokens).

Participants initiated each trial by pressing the spacebar. After the presentation of a token, the text “Press Spacebar if appropriate” appeared on the screen. If participants pressed the spacebar within 2000 ms of hearing a Go token, they were rewarded with a smiley face, a pleasant “ding” sound, and 1 point. Participants were also rewarded if they refrained from pressing the spacebar after hearing a No-go token. However, if they pressed the spacebar within 2000 ms after hearing a No-go token, they were penalized with a sad face, an unpleasant “punch” sound, and no point. Participants were also penalized if they failed to press the spacebar after hearing a Go token. Following each trial, feedback appeared on the screen for 2000 ms. For correct responses, text presented on the screen informed participants that they had earned 1 point (“Congratulations! You have earned 1 point”) and provided a running total of the points acquired (“You have earned a total of X points!”).

In each Go/No-go task, participants completed a pre-training phase using highly discriminable tokens to familiarize them with the task (2 blocks of 10 randomized trials of nonsense words “deet” and “pon” spoken by a female voice with a native Australian English accent). The aim of the pre-training was to familiarize participants with the concept of the Go/No-go task, so the speaker and nonsense words “bon” and “deet” were not in any way related to our stimuli of interest. Pre-training was followed by a training phase (3 blocks of 20 randomized trials of *FAM* /I/ and /ε/, total 60 trials) and a test phase (6 blocks of 20 randomized trials of *FAM* and *NEW*, total 120 trials with 96 *FAM* and 24 *NEW* tokens). *NEW* tokens differed from *FAM* tokens depending on the condition (see Fig 2). Participants were not informed that feedback would not be given for responses to *NEW* tokens or to 25% of responses *FAM* tokens in the test phase. In this way, we obtained 6 responses to each token of *FAM* and *NEW* (12 responses per vowel type per speaker). Because responses of *NEW* tokens were randomly interspersed between *FAM* tokens and were never rewarded or penalized, we obtained data on how participants categorized *NEW* tokens that were not biased by feedback. Data analysis was conducted on these trials in which feedback was not provided (total of 48 trials: 12 *FAM* Go, 12 *FAM* No-go, 12 *NEW* Go, 12 *NEW* No-go).

Our dependent variable was the number of Go responses to *NEW* Go and *NEW* No-go trials in the test phase. Go responses to a *NEW* token suggests that listeners judge it as belonging to the same category as the *FAM* Go stimuli. Not responding (No-go) to a *NEW* token suggests that listeners judge it as belonging to the same category as the *FAM* No-go stimuli. Thus, our dependent variable captures hits, misses, and false alarms, thereby providing a good measure of whether listeners correctly or incorrectly categorized *NEW* test tokens.

#### Statistics

We analyzed the data using a generalized linear mixed model (GLMM) with a binary logistic regression, using the number of Go responses as the dependent variable. The GLMM allows us to fit individual slopes and intercepts for each listener, and to analyze non-normally distributed data with the binary logistic regression, which is suitable for dependent measures such as ours that involve binary categorical responses (i.e. Go or No-go). Stimulus type (*FAM* Go, *FAM* No-go, *NEW* Go, *NEW* No-go), condition (Speaker, Sex, Accent, Accent+Sex), and population (Dutch or AusE) were entered as fixed factors, and participant ID was entered as a random effect in the GLMM. Only higher order interactions that included stimulus type were included as additional fixed factors. Satterthwaite correction for degrees of freedom was applied, as required when performing GLMMs that use pseudolikelihood estimation [53]. For AusE listeners, we first analyzed the effect of linguistic background (monolingual or multilingual) on categorization responses and found no effect (*F*(1,307) = 0.20, *p* = 0.654). For both AusE and Dutch listeners we also analyzed the effect of order of testing, and found that the first condition (e.g. Speaker) did not influenced performance on the second condition (e.g. Sex; *F*(1,312) = 2.48, *p* = 0.116). Thus we did not include these variables as factors in our final model. Statistical analyses were performed in SPSS 21.0.

#### Results & Discussion

**Fig 4:**
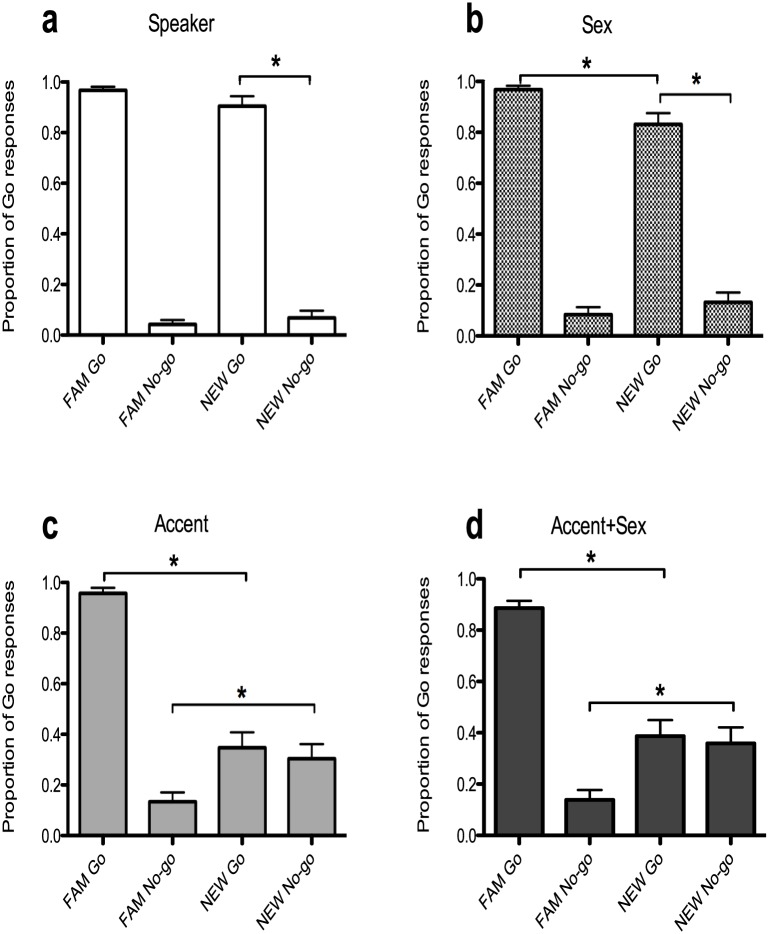
Classification *FAM* and *NEW* tokens by AusE and Dutch listeners (combined) in Experiment 1 when no feedback was provided. Error bars are SEM.

Our analyses demonstrate that listeners could categorize *NEW* vowels in Speaker and Sex conditions, but not in Accent or Accent+Sex conditions (Fig 4). Results of the GLMM showed a significant main effect of stimulus type (*F*(3,329) = 139.69, *p* < 0.001), and significant interactions of population × stimulus type (*F*(3,329) = 7.33, *p* < 0.001) and condition × stimulus type (*F*(9,145) = 9.33, *p* < 0.001). Dutch listeners were better than AusE listeners at classifying *FAM* Go, *FAM* No-go, and *NEW* No-go tokens (*p* < 0.01 for all comparisons with Bonferroni-correction for pairwise comparisons) irrespective of condition (i.e. no significant interaction of population × condition × stimulus type).

To examine the condition × stimulus type interaction, we conducted three simple planned comparisons between: (1) *FAM* Go and *NEW* Go, (2) *FAM* No-go and *NEW* No-go, (3) *NEW* Go and *NEW* No-go. We chose these specific planned comparisons because (1) comparing *FAM* Go to *NEW* Go determines whether inaccurate performance was due to inability to recognize NEW Go tokens as similar to *FAM* Go tokens; (2) comparing *FAM* No-go to *NEW* No-go determines whether inaccurate performance was due to inability to recognize *NEW* No-go tokens as similar to *FAM* No-go tokens; (3) comparing *NEW* Go and *NEW* No-go determines whether listeners were able to discriminate between the vowel categories of a novel speaker. In the Speaker and Sex conditions, Dutch and AusE listeners were able to discriminate between *NEW* vowels (*p* < 0.001), and classified *NEW* tokens as similar to *FAM* tokens (with the exception of Go stimuli in the Sex condition; Fig 4a and 4b). In contrast, Dutch and AusE listeners could not correctly categorize *NEW* tokens in the Accent and Accent+Sex conditions, and treated *NEW* /I/ and /ε/ tokens as different from *FAM* /I/ and /ε/ tokens (*p* < 0.05 for all comparisons; Fig 4c and 4d). Table 1 shows the performance of AusE and Dutch listeners in all four conditions.

**Table 1:** Categorization performance of AusE and Dutch listeners during test trials in Experiment 1. Feedback was not provided for all trials with NEW tokens and for a subset of trials with FAM tokens (used in data analyses).

	Speaker		Sex		Accent		Accent+Sex	
	*Mean*	*SEM*	*Mean*	*SEM*	*Mean*	*SEM*	*Mean*	*SEM*
AusE								
*FAM* Go	0.949	0.015	0.95	0.016	0.877	0.022	0.89	0.028
*FAM* No-go	0.123	0.019	0.125	0.029	0.225	0.036	0.223	0.037
*NEW* Go	0.824	0.041	0.848	0.041	0.398	0.059	0.379	0.061
*NEW* No-go	0.157	0.030	0.219	0.038	0.310	0.055	0.365	0.059
Dutch								
*FAM* Go	0.979	0.017	0.979	0.019	0.986	0.014	0.882	0.052
*FAM* No-go	0.014	0.012	0.056	0.037	0.076	0.041	0.083	0.045
*NEW* Go	0.951	0.040	0.813	0.082	0.299	0.100	0.396	0.112
*NEW* No-go	0.028	0.023	0.076	0.044	0.299	0.099	0.354	0.108

Therefore, our results show that speaker and accent variation are handled differently. In the absence of feedback on their accuracy of adaptation to *NEW* tokens, listeners can adapt to speaker differences but not for accent differences. This applies to even listeners who are familiar with the accent (i.e. Dutch listeners), suggesting that knowledge of the accent is not sufficient for accent adaptation if there is no feedback that listeners can use to judge the accuracy of their accent adaptation.

## Experiment 2

In Experiment 2, we tested whether feedback on the accuracy of *NEW* vowel categorization responses could rescue listeners’ categorization performance of accented vowels. Specifically, AusE listeners were given feedback (correct or incorrect) on their categorization responses to *NEW* tokens in the Speaker, Sex, Accent, and Accent+Sex conditions. We expected that giving feedback to AusE listeners would allow them to correctly categorize the *NEW* vowels in Accent and Accent+Sex conditions which they had previously failed to categorize in Experiment 1.

### Materials and Methods

#### Participants

All participants were adults and provided written informed consent before participating in the experiments. The ethics protocols and written consent procedure for these experiments were approved by the Faculty of Humanities Ethics Committee (University of Amsterdam) and Human Research Ethics Committee (University of Western Sydney).

Forty-four undergraduate students (35 females, 9 males) from the University of Western Sydney participated in Experiment 2 in exchange for course credit (Mean age = 21.93 years, SD = 5.61, Range = 16–45 years). Of these 44, sixteen had previously participated in the categorization task, approximately two weeks prior (the returning participants were collected in a study where performance of humans and birds doing both Experiment 1 and 2 were compared [34]). The remaining 28 participants were naïve to the task. Eighteen participants were monolingual AusE speakers, while 22 spoke one language other than English (i.e. bilingual), and four spoke multiple languages other than English (i.e. multilingual). The additional languages spoken (with the numbers of participants speaking each language indicated in parentheses) were Arabic (10), Assyrian (1), Bengali (1), Cantonese (1), Egyptian (1), Farsi (1), Fijian (1), French (2), German (1), Hindi (2), Macedonian (1), Mandarin Chinese (1), Polish (1), Portuguese (1), Romanian (1), Tagalog (1), Turkish (1), Urdu (1), and Vietnamese (2). All participants had English as their first language and none had prior exposure to Dutch or to any Dutch accent. The participant group who had previously completed the Experiment 1 (i.e. returning participants) was equivalent to the naïve group in terms of age, sex distribution, and language background. Twenty-five participants were assigned to the Speaker and Sex conditions (20 females, Mean age = 21.56 years, SD = 5.56, Range = 17–45 years), and 19 to the Accent and Accent+Sex conditions (15 females, Mean age = 22.42 years, SD = 5.80, Range = 16–38 years).

#### Stimuli

The same stimuli and speaker sets used in Experiment 1 were used in Experiment 2 (see Fig 1).

#### Procedure

We used a modification of the Go/No-go task from Experiment 1: the important differences between Experiments 1 and 2 are the availability of feedback on responses to *NEW* tokens and grouping of test items in Experiment 2. Recall that in Experiment 1, participants completed a pre-training phase, a training phase, and a test phase where *FAM* and *NEW* tokens were intermixed. In Experiment 2, participants completed a pre-training phase (2 blocks of 10 randomized trials of “deet” and “pon”), and two training phases (total 120 trials) where *FAM* and *NEW* tokens were presented separately and not intermixed. The first training phase consisted of 3 blocks of 20 randomized trials of *FAM* /I/ and /ε/ (total 60 trials), similar to Experiment 1. The second training phase consisted of 3 blocks of 20 randomized trials of *NEW* /I/ and /ε/ (total 60 trials). Feedback was provided in both the first and second training phase. Data analysis was conducted on classification performance on the 60 *FAM* trials in the first training phase and the 60 *NEW* trials in the second training phase.

#### Statistics

In order to detect whether listeners may have used feedback to gradually adapt to speaker and accent changes in *NEW* tokens, we examined change in listeners’ performance over time. We divided *FAM* trials and *NEW* trials into blocks of 10 trials and used the proportion correct responses for each block as the dependent variable. Proportion correct responses were calculated as (#Go responses + (#No-go trials—#No-go responses)) / (#Go trials + # No-go trials). We used proportion correct instead of measures such as d-prime because of the small number of trials per block and the high frequency in which hit rates of 1 and false-alarm rates of 0 were observed in our data (~70% of the time). Fewer trials make d-prime estimates less accurate, and corrections for 0 and 1 values also distort the estimate [54].

We conducted a linear mixed model with restricted maximum likelihood (REML) on proportion correct responses with condition (Speaker, Sex, Accent, Accent+Sex), block, participant status (naïve/returning) and the interaction of condition × block and condition × block × status as fixed effects. Participant ID was also included as a random effect. As each listener was tested in two conditions, we first analyzed whether receiving feedback to *NEW* tokens in the first condition would affect classification of *NEW* tokens on the second condition (and hence whether this factor should be included in the linear mixed model). Paired samples t-tests of number of responses to *NEW* tokens in the first condition was not significantly different from the number of responses to *NEW* tokens in the second condition (Condition1 *NEW* Go and Condition2 *NEW* Go *t*(38) = - 1.695, *p* = 0.098; Condition1 *NEW* No-go and Condition2 *NEW* No-go *t*(38) = 0.140, *p* = 0.890). Thus, classifying Dutch/Flemish vowels in the first condition (with feedback) did not significantly influence AusE listeners’ performance on the second condition. Consequently, we did not include the order of conditions as a factor in the linear mixed model.

#### Results and Discussion

**Fig 5:**
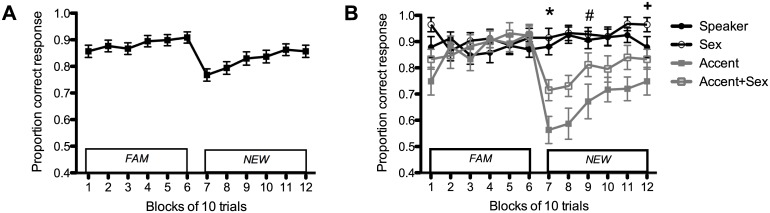
Percent correct categorization responses of AusE listeners in Experiment 2 when feedback was provided. (**A**) Proportion of correct responses pooled across all conditions. (**B**) Proportion of correct responses split by condition. When listeners were switched to NEW (Block 7), accuracy in Accent and Accent+Sex conditions was significantly lower than in Speaker and Sex conditions, as indicated by * (*p* < 0.05). Two blocks after the switch, accuracy in the Accent+Sex condition was no longer significantly different than Speaker, Sex (indicated by #); however, Accent remained significantly lower than Speaker and Sex in that block (*p* = 0.003). Accuracy in the Accent condition was no longer significantly different than Speaker or Accent+Sex in the last block (indicated by +), but was still significantly lower than Sex (*p* = 0.002). Error bars are SEM.

Our findings demonstrated that feedback allowed participants to successfully adapt to accent variation in which they were previously unsuccessful, as they correctly classified *NEW* vowels in Speaker, Sex, Accent, and Accent+Sex conditions (Fig 5). Results showed that all main effects were significant (condition *F*(3,100.48) = 13.04, *p <* 0.001, block *F*(11, 95.63) = 6.98, *p* < 0.001, status *F*(1,39.35) = 5.66, *p* = 0.022). Listeners performed most accurately in the Sex condition and least accurately in the Accent condition (Table 2). They also had the lowest proportion correct responses when they were initially switched to a different speaker (Fig 5A). Returning participants had overall higher accuracy than naïve participants (mean ± SEM of naïve = 0.815 ± 0.019; returning = 0.894 ± 0.026).

**Table 2:** Proportion correct responses in the different conditions.

Condition	Mean	SEM
Speaker	0.891	0.022
Sex	0.923	0.021
Accent	0.767	0.027
Accent+Sex	0.837	0.026

The interaction of condition × block was also significant (*F*(33, 76.56) = 4.95, *p* < 0.001), but the three-way interaction of condition × block × status was not. We focused subsequent analyses on this interaction because it addresses our hypothesis that speaker adaptation occurs automatically, but accent adaptation requires more information (i.e. feedback in our experiment) in order to occur. Specifically, we performed pairwise comparisons with Bonferroni corrections on the significant interaction of condition × block. We did not analyze returning and naïve listeners separately since the interaction term involving status was non-significant. If speaker and accent adaptation both occur automatically and require the same amount of information, then accuracy in the block immediately after they were switched from *FAM* to *NEW* (i.e. Block 7) should be similar across conditions. But if only speaker adaptation is automatic and only accent adaptation requires more information, then accuracy in Speaker and Sex conditions should be greater than in the Accent and Accent+Sex conditions.

We found that performance in the Speaker and Sex conditions was unaffected by the switch to *NEW*, whereas, performance in the Accent and Accent+Sex conditions was significantly affected. Listeners in the Accent and Accent+Sex conditions had significantly lower proportion correct responses than listeners in the Speaker and Sex conditions when they were first switched to *NEW* (*p <* 0.05, Bonferroni-corrected; Fig 5B). In this block, performance on the Accent and Accent+Sex conditions did not significantly differ, and performance on the Speaker and Sex conditions did not significantly differ. Notably, performance on all four conditions was not significantly different in the block preceding the switch (i.e. Block 6; Fig 5B). In the Accent and Accent+Sex conditions, performance on Block 6 preceding the switch was significantly higher than on Block 7 after the switch (*p* < 0.01 for both). In the Speaker and Sex conditions, performance on Block 6 did not significantly differ from Block 7. Therefore, these results support our hypothesis that listeners adapt to speakers automatically, but not accents. The difference between Speaker/Sex and Accent/Accent+Sex conditions was also not likely driven by a ceiling effect because we analyzed the effect of feedback within conditions, i.e. performance on Block 6 (immediately before the switch to *NEW* tokens) compared to performance in Block 7 (immediately after the switch to *NEW* tokens) and found that only performance of listeners in the Accent and Accent+Sex conditions was affected by the switch to *NEW* tokens.

Our results also showed that listeners can use the simple feedback provided in our experiment as additional information to help them eventually adapt to accents. Two blocks after the switch (Block 10), listeners in the Accent+Sex condition performed similarly to listeners in the Speaker and Sex conditions, however, listeners in the Accent condition continued to perform worse than listeners in Speaker and Sex conditions (Fig 5B). Thus, listeners adapted faster in the Accent+Sex condition than the Accent condition.

Altogether, the results of Experiment 2 show that feedback allows listeners to categorize the vowels of a speaker with a different accent, even if they have never had experience with that accent. Feedback regarding the accuracy of adaptation, usually in the form of lexical or semantic information, may be necessary for accent adaptation because it aids perceptual learning, which helps listeners to adjust their phonetic categories in order to be able to appropriately categorize speech sounds (e.g. [55,56]).

## General Discussion

The present study is the first to provide evidence of differential processing of speaker and accent variation by demonstrating that Australian English and Dutch listeners use different methods to handle speaker and accent variation in non-native and native vowels. These results were obtained using vowel stimuli produced by multiple speakers, with non-native listeners with diverse language backgrounds (i.e. many multilingual Australian English listeners), and with native listeners familiar with the accent variation (i.e. Dutch listeners). This indicates that these findings are not due to idiosyncrasies in our stimuli, nor specific to listeners from any particular language background. Specifically, Experiment 1 showed that both Australian English and Dutch listeners performed similarly when confronted with a Speaker/Sex change or an Accent/Accent+Sex change in that they were able to categorize vowels in the Speaker and Sex change conditions, but not in the Accent and Accent+Sex change conditions. Importantly, more opportunities to adapt to Flemish accents does not explain poor accent adaptation, as our Dutch listeners with prior experience with Flemish accents also could not adapt to accents without feedback on the accuracy of their categorization responses. Therefore, our findings strongly support our hypothesis that speaker and accent variation are handled differently, and specifically that speaker/sex adaptation occurs by default whereas accent adaptation does not. The results of Experiment 2 also support our view that listeners can adapt to accented vowels if given access to cues about the accuracy of their adaptation. We found that AusE listeners were able to successfully categorize Flemish accented vowels when they were given simple feedback about the responses (i.e. correct or incorrect) in the Accent and Accent+Sex change conditions. This finding shows that listeners can use simple cues to adapt to speaker and accent differences in vowel production, and there is considerable evidence that adult listeners do use lexical and phonetic cues about whether their phoneme categorizations are correct or incorrect [56–58]. Therefore, our research reaffirms previous work showing that listeners benefit from explicit cues in speech, such as knowledge of talker-specific attributes (e.g. identity, sex, sociolinguistic background) and lexical and semantic content, because it helps them to continually and very rapidly adjust their perceptual categorization of accented speech [17,18,37,38,59,60].

Based on our results, we suggest that speaker and sex may be readily deducible from acoustic-information alone, but accent-related cues are not inherent in the acoustic-phonetic information, which is why contextual information (i.e. semantic and lexical information) and/or feedback on accuracy of vowel categorization is necessary for accent adaptation. The lower accuracy scores in the Accent/Accent+Sex conditions compared to Speaker/Sex therefore suggest that when listeners are given acoustic-phonetic information without contextual information or feedback, the default mechanism is to adapt to the speaker and sex cues available in our vowel stimuli, leading to high accuracy scores in Speaker and Sex conditions, and lower accuracy scores in the Accent and Accent+Sex conditions. Altogether, the findings of Experiment 1 and 2 reinforce our idea that adapting to accents may be a two-step process, first involving automatic and pre-lexical adaptation to speaker differences (as suggested in numerous studies in human adults, infants, and non-human animals [24–27,29–34]) and second, involving the use of explicit cues that allow listeners to adjust the boundaries of their phonetic categories in order to map accented vowels to the correct phonetic categories. These results open up possibilities for future research to discover whether distinct cognitive mechanisms underlie speaker and accent adaptation or whether speaker and accent adaptation are different because they occur at different stages of processing. One reason why accent adaptation may require more information is because adapting to accents is something that listeners have to do in only specific contexts (i.e. when listening to a non-native speaker). On the other hand, listeners must always adapt to speakers, regardless of context (i.e. our auditory systems may be by default tuned to adapt to speaker/sex differences). Consequently, it makes sense that listeners rely on supplementary contextual, lexical, and semantic knowledge in order to adapt to accents.

Our view that listeners handle speaker and accent variation differently are in contrast with exemplar-based theories of speech perception that claim that speaker and accent differences are handled similarly, with accent differences being treated as extreme examples of speaker differences [59,61]. These theories posit that each speech sample is stored as a specific instance/exemplar; exemplars can be tagged with various category labels (e.g. indexical and linguistic labels) that are associated with specific acoustic properties of the speech stimulus [62]. Subsequent speech samples are then compared to stored exemplars, according to whichever category label the listener chooses, and categorized based on similarity to those exemplars [62–66]. Category labels allow listeners to separate auditory properties that are important (i.e. linguistic versus indexical information) and ignore variation in certain dimensions by adjusting attention weights [62]. From the perspective of exemplar-based models, additional contextual information or feedback may be necessary in order to adjust attention weights and shift focus to auditory properties that distinguish accents. We believe, however, that exemplar-based models do not adequately explain our results because the acoustic variability in F1 and F2 between speakers and between accents was comparable (Fig 1), so if listeners were to adapt to speaker differences by comparing *NEW* to *FAM* tokens, then they should also have been able to adapt to accent differences in the same way (note that acoustic cues other than F1 and F2 may have contributed to the difference between *NEW* and *FAM* tokens). We emphasize that this interpretation of our results is not intended to discredit exemplar-based theories and our interpretation that speaker adaptation is automatic while accent adaptation requires more information is not incompatible with exemplar-based theories. Rather, we suggest that by default incoming speech signals activate stored exemplars of speakers with an accent typical of the listener’s native language or local sociolinguistic community (thus allowing speaker adaptation). Note that exemplar models advocate that traces that are acoustically most similar to the incoming speech sample are activated (regardless of accent); however, traces of the listener’s native accent presumably outweigh and are more likely to be activated than those of non-native accents because they likely occur with greater frequency in the listener’s linguistic community. Additional contextual cues may subsequently be needed to trigger activation of stored exemplars of speakers with a different accent that is non-native or foreign. Our suggestion is in line with proposals of hybrid models that incorporate multiple levels of representation from abstractionist models and perceptual learning from exemplar models [67].

In fact, our finding that returning listeners perform better than naïve listeners in Experiment 2 corroborate exemplar-based views that many properties of the acoustic signal of speech are retained, rather than stripped away during vowel category learning. This performance enhancement may result from familiarity with voices, which has been shown to facilitate word recognition [38]. Recall that Experiment 1 did not provide listeners with any feedback, so better categorization of *NEW* tokens in in Experiment 2 by returning listeners could not have resulted from listeners being taught the correct categorization responses in Experiment 1. Alternatively, returning listeners enhanced performance may simply be due to more experience with the task. Future potential investigations into whether voice familiarity may enhance pre-lexical perception of accented vowels should therefore control for practice effects.

One may wonder whether the feedback-mediated improvements in vowel categorization in Experiment 2 actually reflect perceptual learning of accents, prior activation of adaptation strategies, or a simpler form of non-linguistic learning, such as memorizing which sounds correspond to correct or incorrect response options. If listeners memorized mappings, this could have only been done after some experience, but not in the first few trials after hearing *NEW* tokens. Our analysis of Experiment 2 showed that on the first few trials of *NEW* vowels, performance in the Speaker and Speaker+Sex condition was unaffected, whereas performance in the Accent and Accent+Sex conditions dropped significantly and gradually improved over time. Memorization of which *NEW* tokens correspond to correct or incorrect response categories does not explain why listeners in the Speaker and Speaker+Sex conditions maintained high performance while listeners in the Accent and Accent+Sex condition did not, and there is no reason to believe that our listeners in different experimental conditions would have approached the task with different strategies. Lexical feedback—which was intentionally absent from our study—is thought to be the main mechanism by which listeners adapt to accented speech [17]. However, it is not the only way that perceptual learning of accents can occur (since even young children with small lexicons are capable of accent adaptation, e.g. [39,40,68]) and attention to suprasegmental deviations has been proposed to be another way by which listeners can adapt to accents [17]. This suggests that accent adaptation can potentially be achieved by different means, perhaps even by feedback on accuracy during categorization of accented vowels. Future experiments could address this by comparing listeners’ ability to generalize knowledge of accented vowel categories to words, after being exposed to accented vowels with or without feedback. We expect that separate processes for adaptation to speaker and accent also extend to the level of words, where speaker adaptation occurs almost immediately after word onset and accent adaptation occurs close to or after words are completed. The same arguments we implement for vowel adaptation can be used: speaker and sex differences are deducible from acoustic-phonetic information, which allow speaker/sex adaptation to occur rapidly in short speech segments, whereas accent differences are inferred from contextual/lexical information and therefore require more segments and time for lexical access and retrieval.

Another possible interpretation of our findings is that categorization performance is impaired in the Accent and Accent+Sex conditions because detecting a speaker change or sex change is easier than detecting an accent change. Although these explanations may be possible, it is not overtly clear to us how detecting a speaker change would be easier than detecting an accent change, since an accent change also involves a speaker change. At the same time, the opposite may also be possible. That is, detecting different accents may be easier than detecting different speakers. Data from a pre-attentive task suggests that listeners’ brains are more sensitive to accent and gender changes than speaker changes [69]. Infant data also indicate that infants are as equally sensitive to both speaker and accent changes [70]. Together these results suggest that differences between performance to speaker and accent conditions are not likely attributable to difficulty in detecting the accent change, and that the trouble listeners experienced in accent conditions even when feedback was provided may reflect gradual perceptual learning and adjustment of phonetic categories. With regards to sex potentially being more salient than accent, we found that listeners could not adapt to accented vowels even if there was no change in speaker sex, i.e. listeners could adapt to a novel speaker of the same sex with the same accent (Speaker condition) but not a novel speaker of the same sex with a different accent (Accent condition). Hence, differences in saliency between sex and accent do not invalidate our interpretation that adaptation to speaker and sex differences is automatic while adaptation to accents is not.

Interestingly, we found that listeners performed better in the Accent+Sex condition compared to the Accent condition in Experiment 2. We are unsure exactly why this occurs, although one possibility is that the change in speaker sex in the Accent+Sex condition caused *NEW* tokens to sound much more different from *FAM* tokens than the Accent condition (where there was only an accent change). Relatedly, a change in speaker, speaker sex, and accent in the Accent+Sex condition may have been more easily identified than a change in only speaker and accent in the Accent condition. This is because listeners quickly and easily pick up on sex cues [71,72] and there is greater overlap in acoustic properties among speakers of the same sex than between sexes. Thus, a new speaker of a different sex may make it immediately clear to listeners that they should adapt to speaker changes and prompted them to pay closer attention to the *NEW* tokens. This attention may have allowed listeners to adapt to and possibly normalize *NEW* tokens in the Accent+Sex more quickly than *NEW* tokens in the Accent condition.

In conclusion, our study provides strong behavioural evidence that speaker and accent adaptation occur at different stages (and are thus processed differently). Specifically, speaker adaptation appears to be an automatic process whereas accent adaptation may require active processing of additional information such as lexical/semantic feedback to indicate accuracy of speech categorization responses. Our experimental design and use of natural vowel tokens provides the groundwork necessary to conduct further experiments in which the presentation of morphological and sociolinguistic sources of variation is more rigorously controlled. For instance, experiments that present distinctive sex and accent information and track the possible sequential use of the two types of information, or vary either sex or accent information while keeping the other constant would help to verify the existence of a two-step process for speaker and accent normalization and clarify whether additional stages required for accent adaptation may be implemented by the same or different mechanism as speaker adaptation.

## Supporting Information

S1 Data Listener behavior to *NEW* and *FAM* tokens of Experiment 1 and 2 (separated in different sheets). Each listener is uniquely coded in the “Participant ID” column. Numbers in columns labeled *FAM* Go, *FAM* No-go, *NEW* Go, and *NEW* No-go indicate the number of Go responses listeners made (i.e. how many times they pressed “p”). In total, listeners could have made a maximum of 12 Go responses in Experiment 1 and a maximum of 30 Go responses in Experiment 2. Subsequent columns indicate which condition listeners were assigned (“Condition”), their performance on pre-training with “bon” and “deet” tokens (“% correct training”), language background (“ismonolingual”, where monolingual = 1 and multilingual = 0), study population (“population”, where AUS = AusE and DUT = Dutch), and which stimulus set they were assigned (“stimulus set”). Experiment 2 also contains additional columns indicating whether participants were returning or naïve (“participant status”), and the percent correct responses to *NEW* tokens per block of 10 trials.
(XLSX)

S1 Table Table of formant frequencies of vowel stimuli used in Experiments 1 and 2. (DOCX)
