# Supplementary material for: Speaker and Accent Variation Are Handled Differently: Evidence in Native and Non-Native Listeners
Source: PLoS One. 2016 Jun 16;11(6):e0156870. doi: 10.1371/journal.pone.0156870 (PMC4911083; doi:10.1371/journal.pone.0156870)
Supplement: S1 Table — (DOCX) [file pone.0156870.s002.docx]

**Supporting Information**

S1 Table. **Table of formant frequencies of vowel stimuli used in Experiments 1 and 2.**

| Condition | Stimulus type | F1 | F2 | F3 |
| --- | --- | --- | --- | --- |
| speaker | *FAM* | 572 | 1959 | 2861 |
| speaker | *FAM* | 528 | 1925 | 2755 |
| speaker | *NEW* | 572 | 2104 | 2750 |
| speaker | *NEW* | 531 | 2081 | 3067 |
| speaker | *FAM* | 514 | 1935 | 3185 |
| speaker | *FAM* | 471 | 2052 | 3293 |
| speaker | *NEW* | 642 | 2071 | 2836 |
| speaker | *NEW* | 599 | 2064 | 2850 |
| speaker | *FAM* | 394 | 2306 | 2772 |
| speaker | *FAM* | 433 | 2277 | 2772 |
| speaker | *NEW* | 424 | 2288 | 2981 |
| speaker | *NEW* | 384 | 2400 | 3058 |
| speaker | *FAM* | 430 | 2274 | 3083 |
| speaker | *FAM* | 452 | 2329 | 3183 |
| speaker | *NEW* | 424 | 2220 | 2754 |
| speaker | *NEW* | 398 | 2268 | 2756 |
| sex | *FAM* | 572 | 1959 | 2861 |
| sex | *FAM* | 528 | 1925 | 2755 |
| sex | *NEW* | 434 | 1752 | 2569 |
| sex | *NEW* | 481 | 1729 | 2617 |
| sex | *FAM* | 514 | 1935 | 3185 |
| sex | *FAM* | 471 | 2052 | 3293 |
| sex | *NEW* | 384 | 1624 | 2290 |
| sex | *NEW* | 438 | 1650 | 2286 |
| sex | *FAM* | 497 | 1717 | 2453 |
| sex | *FAM* | 489 | 1666 | 2394 |
| sex | *NEW* | 642 | 2071 | 2836 |
| sex | *NEW* | 599 | 2064 | 2850 |
| sex | *FAM* | 521 | 1766 | 2403 |
| sex | *FAM* | 524 | 1762 | 2332 |
| sex | *NEW* | 572 | 2104 | 2750 |
| sex | *NEW* | 531 | 2081 | 3067 |
| sex | *FAM* | 394 | 2306 | 2772 |
| sex | *FAM* | 433 | 2277 | 2772 |
| sex | *NEW* | 356 | 1856 | 2510 |
| sex | *NEW* | 339 | 1895 | 2542 |
| sex | *FAM* | 430 | 2274 | 3083 |
| sex | *FAM* | 452 | 2329 | 3183 |
| sex | *NEW* | 316 | 1774 | 2324 |
| sex | *NEW* | 323 | 1754 | 2330 |
| sex | *FAM* | 363 | 2003 | 2528 |
| sex | *FAM* | 361 | 1818 | 2360 |
| sex | *NEW* | 424 | 2220 | 2754 |
| sex | *NEW* | 398 | 2268 | 2756 |
| sex | *FAM* | 371 | 1963 | 2575 |
| sex | *FAM* | 393 | 1901 | 2476 |
| sex | *NEW* | 424 | 2288 | 2981 |
| sex | *NEW* | 384 | 2400 | 3058 |
| accent | *FAM* | 572 | 1959 | 2861 |
| accent | *FAM* | 528 | 1925 | 2755 |
| accent | *NEW* | 616 | 1780 | 3061 |
| accent | *NEW* | 655 | 1726 | 3140 |
| accent | *FAM* | 642 | 2071 | 2836 |
| accent | *FAM* | 599 | 2064 | 2850 |
| accent | *NEW* | 722 | 1811 | 3028 |
| accent | *NEW* | 722 | 1901 | 3119 |
| accent | *FAM* | 497 | 1717 | 2453 |
| accent | *FAM* | 489 | 1666 | 2394 |
| accent | *NEW* | 523 | 1787 | 2378 |
| accent | *NEW* | 530 | 1759 | 2493 |
| accent | *FAM* | 434 | 1752 | 2569 |
| accent | *FAM* | 481 | 1729 | 2617 |
| accent | *NEW* | 580 | 1721 | 2719 |
| accent | *NEW* | 594 | 1660 | 2707 |
| accent | *FAM* | 394 | 2306 | 2772 |
| accent | *FAM* | 433 | 2277 | 2772 |
| accent | *NEW* | 520 | 1854 | 2942 |
| accent | *NEW* | 582 | 1934 | 2994 |
| accent | *FAM* | 424 | 2220 | 2754 |
| accent | *FAM* | 398 | 2268 | 2756 |
| accent | *NEW* | 503 | 2036 | 3001 |
| accent | *NEW* | 534 | 1965 | 3086 |
| accent | *FAM* | 363 | 2003 | 2528 |
| accent | *FAM* | 361 | 1818 | 2360 |
| accent | *NEW* | 411 | 1822 | 2453 |
| accent | *NEW* | 365 | 1849 | 2481 |
| accent | *FAM* | 356 | 1856 | 2510 |
| accent | *FAM* | 339 | 1895 | 2542 |
| accent | *NEW* | 426 | 1836 | 2711 |
| accent | *NEW* | 450 | 1788 | 2696 |
| accent+sex | *FAM* | 572 | 1959 | 2861 |
| accent+sex | *FAM* | 528 | 1925 | 2755 |
| accent+sex | *NEW* | 580 | 1721 | 2719 |
| accent+sex | *NEW* | 594 | 1660 | 2707 |
| accent+sex | *FAM* | 642 | 2071 | 2836 |
| accent+sex | *FAM* | 599 | 2064 | 2850 |
| accent+sex | *NEW* | 520 | 1543 | 2366 |
| accent+sex | *NEW* | 521 | 1560 | 2566 |
| accent+sex | *FAM* | 497 | 1717 | 2453 |
| accent+sex | *FAM* | 489 | 1666 | 2394 |
| accent+sex | *NEW* | 674 | 1766 | 2916 |
| accent+sex | *NEW* | 683 | 1742 | 2925 |
| accent+sex | *FAM* | 434 | 1752 | 2569 |
| accent+sex | *FAM* | 481 | 1729 | 2617 |
| accent+sex | *NEW* | 616 | 1780 | 3061 |
| accent+sex | *NEW* | 655 | 1726 | 3140 |
| accent+sex | *FAM* | 394 | 2306 | 2772 |
| accent+sex | *FAM* | 433 | 2277 | 2772 |
| accent+sex | *NEW* | 426 | 1836 | 2711 |
| accent+sex | *NEW* | 450 | 1788 | 2696 |
| accent+sex | *FAM* | 424 | 2220 | 2754 |
| accent+sex | *FAM* | 398 | 2268 | 2756 |
| accent+sex | *NEW* | 452 | 1744 | 2541 |
| accent+sex | *NEW* | 428 | 1729 | 2576 |
| accent+sex | *FAM* | 363 | 2003 | 2528 |
| accent+sex | *FAM* | 361 | 1818 | 2360 |
| accent+sex | *NEW* | 519 | 1831 | 2905 |
| accent+sex | *NEW* | 490 | 1811 | 2847 |
| accent+sex | *FAM* | 356 | 1856 | 2510 |
| accent+sex | *FAM* | 339 | 1895 | 2542 |
| accent+sex | *NEW* | 520 | 1854 | 2942 |
| accent+sex | *NEW* | 582 | 1934 | 2994 |
